# Serum interleukin-6 and tumor necrosis factor-α are associated with early graft regeneration after living donor liver transplantation

**DOI:** 10.1371/journal.pone.0195262

**Published:** 2018-04-12

**Authors:** Min Suk Chae, Kwang Uck Moon, Hyun Sik Chung, Chul Soo Park, Jaemin Lee, Jong Ho Choi, Sang Hyun Hong

**Affiliations:** Department of Anesthesiology and Pain Medicine, Seoul St. Mary’s Hospital, College of Medicine, The Catholic University of Korea, Seoul, Republic of Korea; Texas A&M University, UNITED STATES

## Abstract

**Background:**

Liver graft regeneration is orchestrated by specific and sequential stimuli, including hepatocyte growth factors, cytokines, and catecholamines. We evaluated the association between preoperative serum cytokines and early liver graft regeneration in human living donor liver transplantation (LDLT).

**Patients and methods:**

We retrospectively reviewed the data of adult patients who underwent LDLT from January 2010 to December 2014. Serum cytokines, including interleukin (IL)-2, 6, 10, 12, 17, interferon (IFN)-γ and tumor necrosis factor (TNF)-α were measured in the recipients 1 day before surgery and on postoperative day (POD) 7. Liver graft volume was estimated using abdominal computed tomography images of the donors and recipients.

**Results:**

In total, 226 patients were analyzed in this study. Median preoperative levels of serum cytokines were as follows: IL-2, 0.1 (0.1–1.6) pg/mL; IL-6, 7.3 (0.1–30.2) pg/mL; IL-10, 0.5 (0.1–11.0) pg/mL; IL-12, 0.1 (0.1–0.1) pg/mL; IL-17, 2.0 (0.1–16.4) pg/mL; IFN-γ, 3.2 (0.1–16.0) pg/mL; and TNF-α, 9.8 (5.4–17.9) pg/mL. Higher preoperative serum levels of IL-6, IL-10, and TNF-α, dichotomized at the median, were associated with increased relative liver volumes by POD 7. Multivariate analysis revealed that higher levels of serum IL-6 and TNF-α were independently associated with increased graft volume during the first 1 week after LDLT, based on the lower levels of those cytokines.

**Conclusions:**

IL-6 and TNF-α were important mediators of the success of early graft regeneration in patients who underwent LDLT.

## Introduction

Sufficient liver graft regeneration is important in patients who undergo living donor liver transplantation (LDLT) because partial liver grafts are used [1]. Partial liver grafts are required to meet the metabolic burden of recipients with end-stage liver disease (ESLD), and insufficient graft mass is associated with graft dysfunction, leading to poor graft or recipient survival in patients who underwent LDLT [2]. Various clinical factors, including graft size and quality, portal circulation, and immunological factors, affect the degree of postoperative graft regeneration in LDLT [1,3–5].

Cytokines are important proteins with multifunctional roles in immunological processes and are expressed by various inflammatory cells, such as macrophages, B lymphocytes, T lymphocytes and fibroblasts. Pro-inflammatory cytokines include interleukins (IL)-2, 6, 12, 17, interferon (IFN)-γ, and tumor necrosis factor (TNF)-α; and IL-10 is one of the major anti-inflammatory cytokines [6]. Patients with ESLD experience a severe inflammatory response with dysregulation between pro- and anti-inflammatory cytokines in diseased livers. The dysregulation of these cytokines plays essential roles in the development of various inflammatory liver diseases that result in liver cirrhosis [7–10]. In addition, the development of graft rejection after liver transplantation (LT) is associated with the expression of these cytokines, including IL-2, 6, 10, 12, 17, IFN-γ, and TNF-α [11–16].

Hepatocytes in healthy animal livers are in the quiescent G0 phase of the cell cycle; however, after liver tissue loss, such as partial hepatectomy, remnant hepatocytes are stimulated to re-enter the proliferative phases of the cell cycle. Liver regeneration occurs within minutes to hours after liver tissue injury and involves all mature parenchymal and nonparenchymal cells, including hepatocytes, biliary epithelial cells, sinusoidal endothelial cells, Kupffer cells, and stellate cells [17]. This graft regeneration process is orchestrated by specific and sequential stimuli, including hepatocyte growth factors, cytokines, and norepinephrine [18]. Particularly, specific cytokines, including interleukin (IL)-6 and tumor necrosis factor (TNF)-α, play an important role in the liver regeneration process [19,20].

The association between cytokines and liver regeneration has not been sufficiently reported in the human LDLT setting. In the present study, we evaluated the association between preoperative serum cytokine levels and early liver graft regeneration after LDLT. In addition, we describe changes in serum cytokine levels between preoperative day 1 and postoperative day (POD) 7.

## Patients and methods

### Study population

Between January 2010 and December 2014, 260 adult patients (age ≥ 19 years) with ESLD underwent LDLT at St. Mary’s Hospital in Seoul, Republic of Korea. We retrospectively reviewed the hospital electronic medical records of the recipients and donors. Recipients who underwent elective and regular LDLT surgery were included in this study; however, recipients who underwent perioperative hemodialysis, including continuous renal replacement therapy (CRRT), and ABO incompatible LDLT, which require plasmapheresis, were excluded because hemodialysis and plasmapheresis eliminate cytokines from blood circulation [21–25]. This study was approved by the Institutional Review Board of Seoul St. Mary’s Hospital Ethics Committee (KC17RISI0001). The requirement for informed consent was waived due to the retrospective study design.

### Surgery and anesthesia

A piggyback surgical technique was performed, using a modified right hepatic lobe with middle hepatic vein reconstruction according to our hospital surgical protocol [26]. Hepatic vessels, including the hepatic vein, portal vein, and hepatic artery were subsequently anastomosed, followed by reconstruction of the biliary duct. After the hepatic vascular anastomosis, patency of the vessels was evaluated by Doppler sonography.

Details of the anesthetic method are described in our previous study [27]. In summary, balanced anesthesia was applied under multiple monitoring modes. Any patient in an unstable hemodynamic condition was treated by fluid resuscitation and inotrope administration, as appropriate. Blood products, such as packed blood cells, fresh frozen plasma, cryoprecipitate, and platelets, were transfused under laboratory-defined parameters.

### Immunosuppression

Patients who were scheduled for LDLT were administered an immunosuppression regimen, including a calcineurin inhibitor (i.e tacrolimus or cyclosporine), mycophenolate mofetil (MMF), prednisolone, and basiliximab (IL-2 receptor blocker), according to the LDLT immunosuppression protocol [28]. The trough level of tacrolimus was kept between 7 and 10 ng/mL for the first month after LDLT and was subsequently reduced to 5–7 ng/mL. The trough level of cyclosporine was sustained at 100–150 ng/mL for the first month after LDLT, and serially decreased to 50–100 ng/mL thereafter. Prednisolone was progressively tapered within the first month after LDLT, and MMF was postoperatively tapered between 3 and 6 months. Basiliximab was administered on the day before LDLT surgery and on POD 4.

### Serum cytokine measurement

Serum cytokines, including IL-2, IL-6, IL-10, IL-12, IL-17, IFN-γ, and TNF-α were measured in recipients 1 day before surgery and on POD 7. A blood sample was collected using a sterile technique into test tubes (BD Vacutainer, K2 EDTA; Becton, Dickinson and Company, Franklin Lakes, NJ, USA). All blood samples were transported to the laboratory in an ice-filled box, centrifuged (1,500 rpm for 10 min at 4°C), frozen at −70°C, and stored until analysis. Serum cytokine levels were measured using sandwich enzyme-linked immunosorbent assays and a human 25-plex antibody bead kit (Invitrogen Corp., Camarillo, CA, USA). Data were assessed with the LuminexTM detection system (200TM; Luminex Corp., Austin, TX, USA).

### Liver volume measurement

**Fig 1:**
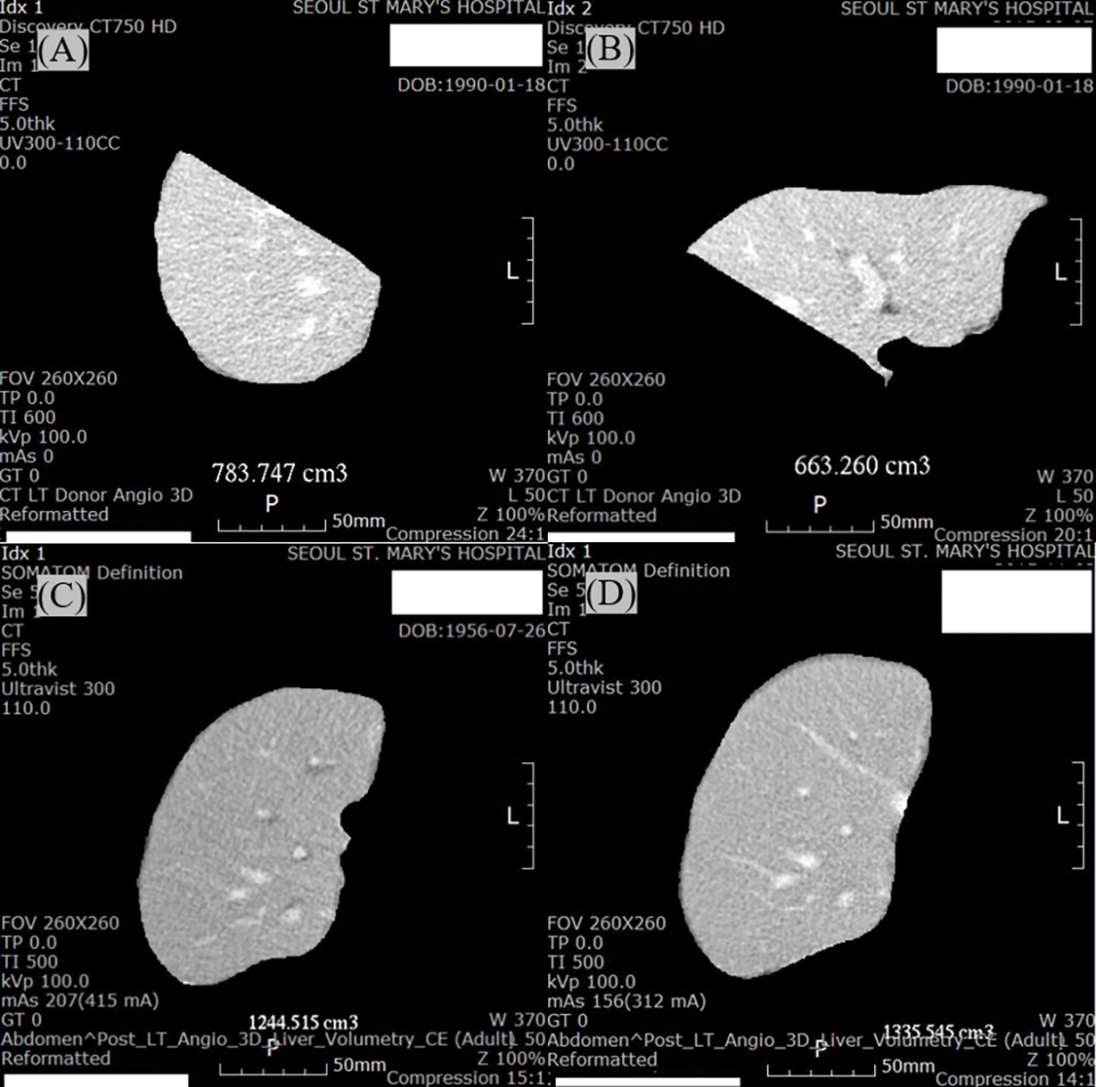
Volumetric computed tomography images in the right (A) and left (B) lobes of a donor preoperatively, and in the right lobe of a liver graft on postoperative days 7 (C) and 21 (D) in a patient who underwent a living donor liver transplantation.

Liver graft volume was estimated using abdominal computed tomography (CT) images of the donors and recipients, which were assessed by experienced radiologists using volumetric software (AW VolumeShare 4; General Electric Healthcare, Chicago, IL, USA). Volumetric CT images of the donors were measured preoperatively to identify total liver volume and right lobe volume, and volumetric CT images of recipients were assessed postoperatively to determine liver graft volumes on PODs 7 and 21 (Fig 1). Absolute liver volume (ALV) was estimated in addition to liver graft volume (mL). Relative liver volume (RLV) was characterized as the ratio between the estimated graft volume and standard liver volume (SLV) (SLV = 1,072.8 × body surface area [BSA] − 345.7; BSA = weight [kg] 0.425 × height [cm] 0.725 × 0.007184) [29].

### Preoperative clinical findings in recipients and donors

Preoperative recipient parameters were age, gender, body mass index (BMI), model for end-stage liver disease (MELD) score, etiology, and laboratory values (hematocrit, sodium, white blood cell [WBC] count, C-reactive protein [CRP], platelets, total bilirubin, international normalized ratio [INR], aspartate aminotransferase [AST], alanine aminotransferase [ALT], and albumin). Preoperative donor parameters were age, gender, BMI, and steatosis.

### Postoperative acute cellular rejection in recipients

Acute cellular rejection within 90 days after LDLT was detected as elevations in liver enzyme levels (i.e., total bilirubin, INR, AST and ALT) with clinical symptoms (fever and jaundice) and confirmed by liver biopsy using a rejection activity index (RAI) ≥ 3 points, which was estimated by experienced pathologists according to the Banff scheme [30].

### Statistical analysis

Continuous data are expressed as medians with interquartile range (IQR), and categorical data are expressed as numbers (proportions). Correlations between preoperative serum cytokine profiles and preoperative clinical recipient findings were analyzed using Spearman’s method. Preoperative serum cytokine levels were compared according to gender using the Mann-Whitney *U* test; etiology and MELD score on a 10 point scale using the Kruskal-Wallis test with a *post-hoc* analysis. Preoperative serum cytokine levels were compared based on acute cellular rejection after surgery using the Mann-Whitney *U* test. Postoperative changes in liver graft volumes were analyzed by one-way repeated-measures analysis of variance. The differences in liver graft volumes and serum cytokine levels between two time points were assessed using the Wilcoxon signed-rank test. The associations between preoperative serum cytokine levels, dichotomized at the median, and changes in liver graft volumes from preoperative day 1 to POD 7 were evaluated by univariate linear regression analysis. Potentially significant factors (*p* < 0.1) in the univariate analysis were entered into a multivariate linear regression analysis. All tests were two-sided, and a *p*-value < 0.05 was deemed significant. Statistical analyses were performed using SPSS for Windows software (ver. 24.0; SPSS Inc., Chicago, IL, USA).

## Results

In total, 34 patients were excluded due to incomplete and inappropriate data, including volumetric CT images (16 patients); and serum cytokine measurements owing to hemodialysis (14 patients) and plasmapheresis due to ABO incompatible LDLT (4 patients). Therefore, 226 patients were analyzed in this study. The median age of the recipients was 53 years (range: 48–59 years), and male gender was predominant (70.4%) (Table 1). The median MELD score was 15 points (range: 9–23 points). Hepatitis B virus (61.1%) was the most common etiology for LDLT followed by alcohol use (19.5%), drugs and toxins (6.6%), hepatitis C virus (5.3%), and hepatitis A virus (2.2%). The median age of the donors was 32 years (range: 25–43 years), and male gender was predominant (57.1%). Median BMI was 23.0 kg/m2 (range: 21.1–25.4 kg/m2), and median percentage of steatosis was 3.0% (range: 0.0–5.0%).

**Table 1:** Preoperative clinical findings in recipients and donors of living donor liver transplantation.

N	226
**Recipient finding**	
Age (years)	53 (48–59)
Gender (male/female)	159/67
Body mass index (kg/m^2^)	24.2 (22.0–26.4)
Model for end-stage liver disease score (point)	15 (9–23)
*Etiology*	
Alcohol	44 (19.5%)
Hepatitis A	5 (2.2%)
Hepatitis B	138 (61.1%)
Hepatitis C	12 (5.3%)
Drug & toxin	15 (6.6%)
Cryptogenic hepatitis	12 (5.3%)
*Laboratory value*	
Hematocrit (%)	29.6 (25.2–35.8)
Sodium (mEq/L)	139 (135–142)
White blood cell count (x10^9^/L)	4.0 (2.7–6.2)
C-reactive protein (mg/dL)	0.4 (0.1–1.2)
Platelet count (x10^9^/L)	63.5 (45.8–108.3)
Total bilirubin (mg/dL)	2.1 (0.9–7.1)
International normalized ratio	1.4 (1.2–1.9)
Aspartate aminotransferase (U/L)	43 (31–77)
Alanine aminotransferase (U/L)	31 (22–48)
Albumin (mg/dL)	3.0 (2.7–3.5)
**Donor finding**	
Age (years)	32 (25–43)
Gender (male/female)	129/97
Body mass index (kg/m^2^)	23.0 (21.1–25.4)
Steatosis (%)	3.0 (0.0–5.0)

Values are expressed as number (portions) and median (interquartile range).

A few preoperative serum cytokines were correlated with preoperative clinical recipient findings (S1 Table). Preoperative serum IL-6 level was moderately correlated with preoperative CRP level, and was weakly correlated with preoperative MELD score and levels of hematocrit, sodium, WBC, total bilirubin, INR, AST, ALT and albumin. Preoperative serum IL-10 level was weakly correlated with preoperative MELD score and levels of hematocrit, WBC, CRP, total bilirubin, AST and ALT. Preoperative serum TNF-α level was weakly correlated with preoperative MELD score and levels of hematocrit, sodium, WBC, CRP, and albumin. Preoperative serum IL-2 level was weakly correlated with preoperative serum sodium level.

No differences in preoperative serum cytokine levels were detected according to gender (S2 Table) or etiology (S3 Table), respectively. However, preoperative serum IL-6, 10, and TNF-α levels increased as MELD score increased by 10 points (S4 Table).

Fifty nine recipients (26.1%) suffered from acute cellular rejection after surgery, and preoperative serum cytokine levels were not different according to the development of acute cellular rejection (S5 Table).

**Fig 2:**
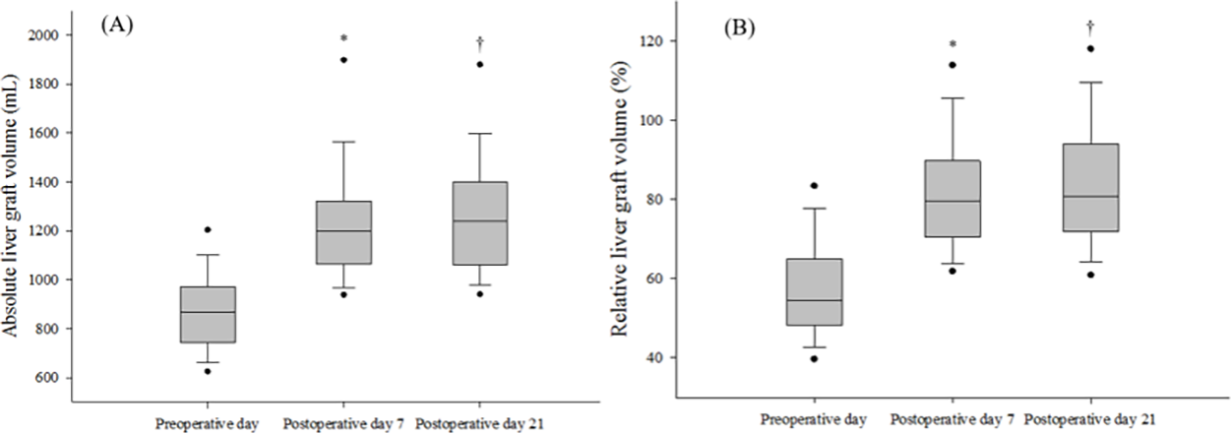
Changes in (A) absolute liver graft volumes (mL), and (B) relative liver graft volumes (%), preoperatively versus days 7 and 21 postoperatively, in patients who underwent living donor liver transplantation. The box plots show the median (line in the middle of the box), interquartile range (box), 5^th^ and 95^th^ percentiles (whiskers), and outliers (dots). *p < 0.001, preoperatively *vs*. postoperative day 7. †*p* < 0.01, postoperative day 7 *vs*. day 21.

**Fig 3:**
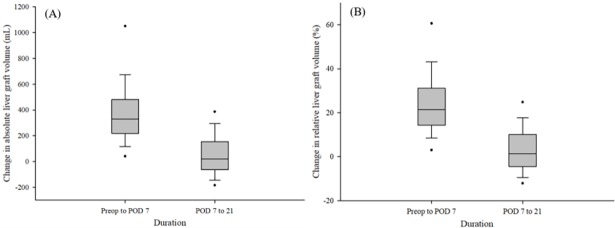
Degrees of changes in (A) absolute liver graft volume (mL) and (B) relative liver graft volume (%) between preoperative day and postoperative day 7; and postoperative days 7 and 21 in patients who underwent living donor liver transplantation. The box plots show the median (line in the middle of the box), interquartile range (box), 5^th^ and 95^th^ percentiles (whiskers), and outliers (dots).

The median absolute liver graft volume was 866.9 mL (range: 739.4–972.3 mL) preoperatively; 1,194.3 mL (1,051.9–1,316.3 mL) on POD 7; and 1,232.8 mL (1,061.2–1,398.1 mL) on POD 21 (Fig 2). The median relative liver graft volume was 54.4% (48.0–64.9%) preoperatively; 78.8% (69.1–89.4%) on POD 7; and 79.9% (71.6–93.6%) on POD 21. In addition, median SLV was 1,540.2 mL (1,386.7–1,644.4 mL). Both ALV and RLV significantly increased from the time of transplantation to POD 7, and then increased from POD 7 to 21. The median change in ALV was 320.5 mL (212.5–475.9 mL) preoperative day to POD 7, and 17.7 mL (−77.6–147.3 mL) from POD 7 to 21 (Fig 3). The median change in RLV was 20.9% (13.5–30.8%) from preoperative day 1 to POD 7, and 1.1% (−5.0–9.6%) from POD 7 to 21.

Serum levels of IL-6, IL-17, and IFN-γ decreased significantly during the first week after surgery; however, serum IL-10 levels increased during that period (Table 2).

**Table 2:** Changes in serum cytokine levels between preoperative day 1 and postoperative day 7 in patients undergoing living donor liver transplantation.

Serum cytokine level (pg/mL)			
	*Preoperative day*	*Postoperative day 7*	*p*
Interleukin-2	0.1 (0.1–1.6)	0.3 (0.1–3.0)	0.133
Interleukin-6	7.3 (0.1–30.2)	4.1 (0.1–20.4)	0.007
Interleukin-10	0.5 (0.1–11.0)	15.9 (4.0–36.2)	0.000
Interleukin-12	0.1 (0.1–0.1)	0.1 (0.1–0.1)	0.500
Interleukin-17	2.0 (0.1–16.4)	0.6 (0.1–6.7)	0.000
Interferon-γ	3.2 (0.1–16.0)	1.7 (0.1–7.9)	0.000
Tumor necrosis factor-α	9.8 (5.4–17.9)	9.6 (5.6–13.9)	0.064

Values are expressed as number (portions) and median (interquartile range).

Higher preoperative levels of serum IL-6, IL-10, and TNF-α, dichotomized at the median, were associated with increased RLVs by POD 7 (Table 3). Multivariate analysis revealed that higher levels of serum IL-6 and TNF- α were independently associated with increases in RLVs during the first week after LDLT, based on the lower levels of those cytokines.

**Table 3:** Association between preoperative serum cytokine levels (dichotomized at the median) and an increased estimated graft volume to standard liver volume ratio from preoperative day 1 to postoperative day 7 in patients undergoing living donor liver transplantation.

		Univariate linear regression			Multivariate linear regression		
Serum cytokine profile (pg/mL)	Median	*β*	95% CI	*p*	*β*	95% CI	*p*
Interleukin-2	0.1	0.335	-3.524–4.195	0.864			
Interleukin-6	7.3	5.463	1.805–9.120	0.004	4.560	0.826–8.293	0.017
Interleukin-10	0.5	4.595	0.915–8.274	0.015			
Interleukin-12	0.1	-1.472	-6.776–3.832	0.585			
Interleukin-17	2.0	0.412	-3.330–4.155	0.828			
Interferon-γ	3.2	1.059	-2.678–4.795	0.577			
Tumor necrosis factor-α	9.8	4.894	1.222–8.565	0.009	3.817	0.087–7.547	0.045

CI, confidence interval

## Discussion

The main finding of the present study was that the IL-6 and TNF-α were important cytokines for early liver graft regeneration in a human LDLT setting. Preoperatively higher levels of serum IL-6 and TNF-α were associated with early liver graft regeneration after LDLT. In addition, serum IL-10 level increased from the preoperative day to POD 7; however, serum IL-6, 17, and IFN-γ levels decreased. Serum TNF-α level did not change during the first week after LDLT.

In this study, partial liver grafts vigorously regenerated during the first week after LDLT. Although the increase in graft regeneration was gradually attenuated thereafter, regeneration continued during the third week after surgery. A study by Marcos *et al*. [31] suggested that graft regeneration, as assessed by volumetric magnetic resonance imaging, occurred rapidly during the first week after living right lobe liver transplantation, and no differences were observed in the time courses of donors and recipients. The underlying process causing rapid liver regeneration during the early postoperative period remains uncertain. Another study by Olthoff *et al*. [4] reported that liver tissue mass is steadily and continuously restored during the first 3 months after LDLT; however, it did not reach complete liver mass recovery.

Many studies have suggested that IL-6 is a key cytokine for inducing liver regeneration after partial hepatectomy and maintaining hepatic homeostasis, which is involved in the acute-phase response, hepatoprotection (i.e., anti-necrosis and anti-apoptosis effects), and hepatocyte mitogenesis [32–34]. In IL-6 ^-/-^ and CCAAT enhancer binding protein β/nuclear factor-IL-6 (a protein involved in triggering of the G0/G1 phase transition) ^-/-^ knockout mice models, intracellular receptors of IL-6 for liver regeneration are evident. Mice studies show that an abnormal hepatocyte G1 phase results in a decreased rate of DNA synthesis, and eventually the proliferative ability of hepatocytes is reduced after partial hepatectomy [18]. In another IL-6 ^-/-^ animal model, exogenous administration of IL-6 resulted in augmentation of hepatocyte DNA synthesis and recovery of enhanced hepatocyte proliferation [35]. After IL-6 is secreted by Kupffer cells in the liver, it binds to its receptor near hepatocytes and induces intracellular signaling via Janus kinase to activate the mitogen-activated protein kinase pathway and the signal transducer and activator of transcription (STAT) 3 pathway. This process results in proliferation and differentiation of hepatocytes [36,37].

In a TNF-receptor-1 knockout model, TNF-α also contributed to the liver regeneration process after partial hepatectomy [19]. A TNF-α antibody treatment before partial hepatectomy results in inhibited liver regeneration, triggered by changes in Jun kinase, c-Jun mRNA, and nuclear AP1 activity. This finding supports that TNF-α allows hepatocytes to become sensitive to mitogens and facilitates liver regeneration [38]. Circulating TNF-α binds to its receptor on Kupffer cells in the liver, which causes enhanced IL-6 transcription through the nuclear factor (NF)-κB pathway. TNF-α and IL-6 stimulate hepatocyte proliferation by increasing activation of signal transducers and the STAT 3 pathway [17]. In addition, the NF-κB pathway, which is regulated by TNF-α, is associated with the anti-apoptotic response during liver regeneration [39].

Remnant liver tissue rapidly regenerates during the first 2 weeks in human living donors after a right lobe hepatectomy, and plasma biomarker levels, including IL-6, TNF-α, and hepatocyte growth factor, are upregulated during the early postoperative period [40]. The present study shared similar findings to those of previous studies [1,17,20,32,34,38,40] in that IL-6 and TNF-α were closely associated with liver regeneration after liver tissue loss or injury. Measuring systemic circulating IL-6 and TNF-α levels in recipients scheduled for LDLT may be helpful to determine whether partial liver grafts regenerate during the early period after LDLT.

Serum TNF-α levels remained unchanged in our study patients between the preoperative day and POD 7, and this may be one of the reasons for an increase in liver graft size after LDLT. TNF-α is an important mediator modulating inter- and intracellular signaling during liver graft regeneration [18,19,38,39]. Although our study showed a similar trend in serum TNF-α levels compared to that of Sasturkar *et al*.[40], we could not evaluate peak and trough levels of serum TNF-α during vigorous graft regeneration under a postoperative immunosuppressive condition. In addition, a previous study suggested that high levels of TNF-α during the postoperative period were correlated with the development of graft rejection in patients who underwent LT [41]. Although further study is required to determine the TNF-α cut-off levels between acute graft rejection and early graft regeneration under specific immunosuppressive regimens in the LT setting, our study identified TNF-α as a predictive marker of liver graft regeneration during the early postoperative period in human LDLT.

This study suggests that serum IL-10 levels increased during the first week after surgery; however, serum levels of IL-6, 17, and IFN-γ decreased after surgery. IL-10 is derived from macrophages and T cells and has potent anti-inflammatory features. Plasma levels of IL-10 markedly increase after graft reperfusion and reach the peak level after LT [42]. In an animal LT study, donor pretreatment with IL-10 or IL-10 administration during graft reperfusion causes significant reductions in cold ischemia reperfusion injury and levels of T cells and macrophage-dependent cytokines, such as TNF and IFN-γ [43]. IL-10^-/-^ mice after partial hepatectomy show higher levels of pro-inflammatory cytokines (i.e., IL-6, TNF-α, and IFN-γ) and inflammatory markers of macrophages (F4/80) and monocytes (CCR2) in the liver and blood compared with those of wild-type mice. The remnant liver in IL-10^-/-^ mice regenerates more vigorously than that in wild-type mice according to a BrdU incorporation assay, which is associated with increased levels of IL-6 and STAT3 [44]. Pro-inflammatory cytokine levels, including IL-6 and IFN-γ, increased during the postoperative period in a previous living donor study [40]. However, because recipients who underwent LDLT are administered immunosuppressive drugs postoperatively to avoid acute graft rejection, serial changes in serum cytokine levels may be different between recipients and donors. High levels of IL-6, 17, and IFN-γ are associated with acute graft rejection in liver transplant recipients [12,14–16]. Therefore, we speculated that immunosuppressants may decrease serum levels of IL-6, 17, and IFN-γ after LDLT; thus, further study is needed to investigate the association between liver graft regeneration and serum cytokine levels under specific immunosuppressive conditions.

The underlying process explaining why higher preoperative serum IL-6 and TNF-α levels affected early postoperative regeneration of transplanted healthy grafts was not clarified in this study. Serum levels of inflammation related cytokines, including IL-6, 10, and TNF-α, tend to increase with the MELD score, which is related to the severity of liver disease [45]. The MELD score increased by 10 points, because the score consists of total bilirubin, creatinine and INR, but no inflammatory markers [46]; thus, the severity of the inflammatory condition in patients with ESLD is not directly measured using the MELD score. We speculated that patients with a higher MELD score suffered from severe liver disease related to severe inflammation [47] than those with a lower score. Further study is required to investigate the relationship between inflammatory cytokines, MELD score and early graft regeneration in the LDLT setting.

Because systemic circulating cytokines were evaluated in this study, we excluded patients who underwent interventions that influenced serum cytokine levels. In patients who suffered from sepsis and acute renal failure, continuous veno-venous hemofiltration with dialysis removed TNF-α and IL-1β from systemic circulation [25]. In severely burned patients with sepsis, plasma levels of endotoxin and cytokines, including TNF-α, IL-1β, IL-6, and IL-8 after CRRT were significantly reduced based on those before the therapy [24]. Therefore, we considered that plasma exchange interventions, such as CRRT and plasmapheresis, decreased the accuracy of the serum cytokine levels.

Our study had several limitations. First, because cytokines within liver graft tissues were not measured using liver biopsies, the direct effect of cytokines on graft regeneration could not be evaluated. Second, because only a few cytokines were analyzed in our study, further studies are required to ascertain the specific effect of different cytokines on postoperative graft regeneration in human LDLT. However, because we focused on serum cytokines related to liver inflammation [7–10] and graft rejection [11–16], interactions between pro-inflammatory cytokines (IL-2, -6, -12, -17, IFN-γ, and TNF-α) and anti-inflammatory cytokines (IL-10) were clarified in the LDLT setting. In addition, we determined serum cytokines related to graft regeneration [18,19] because partial grafts were transplanted in patients who underwent LDLT. Third, although a previous study reported that changes in serum cytokine levels occur during the early postoperative period in living donors who undergo a right lobe hepatectomy [40], we could not evaluate peak and trough levels of serum cytokines during the rapid and vigorous graft regeneration after LDLT under specific immunosuppressive conditions. However, our study identified IL-6 and TNF-α as predictive markers to regenerate the grafts during the early postoperative period. Finally, we cannot suggest preoperative cut-off levels of serum IL-6 or TNF-α to predict the degree of postoperative graft regeneration.

In conclusion, IL-6 and TNF-α are important mediators of early graft regeneration success in patients who underwent LDLT. Preoperatively higher levels of serum IL-6 and TNF-α were associated with greater regeneration of the transplanted healthy graft during the first week after LDLT. To date, many animal studies have investigated the role of IL-6 and TNF-α in liver graft regeneration; however, this study is the first to describe the association between serum cytokines and graft regeneration in a human LDLT setting. Further human studies on specific mediators of graft regeneration could be helpful to guide therapeutic intervention and improve graft regeneration in the context of insufficiently sized liver grafts.

## Supporting information

S1 Table Correlations of preoperative serum cytokine profiles with preoperative clinical recipient findings in living donor liver transplantation. (DOCX)

S2 Table Comparisons of preoperative serum cytokine levels according to gender in patients who underwent living donor liver transplantation. (DOCX)

S3 Table Comparisons of preoperative serum cytokine levels according to etiology in patients who underwent living donor liver transplantation. (DOCX)

S4 Table Comparisons of preoperative serum cytokine levels according to model for end-stage liver disease (MELD) score in patients who underwent living donor liver transplantation. (DOCX)

S5 Table Comparisons of preoperative serum cytokine levels according to acute cellular rejection development after living donor liver transplantation. (DOCX)

## Abbreviations

ALT: alanine aminotransferase
ALV: absolute liver volume
AST: aspartate aminotransferase
BMI: body mass index
BSA: body surface area
CRRT: continuous renal replacement therapy
ESLD: end-stage liver disease
IFN: interferon; IL, interleukin
LDLT: living donor liver transplantation
MELD: model for end-stage liver disease
MMF: mycophenolate mofetil
POD: postoperative day
RAI: rejection activity index
RLV: relative liver volume
STAT: signal transducer and activator of transcription
TNF: tumor necrosis factor
