# Supplementary material for: Serum interleukin-6 and tumor necrosis factor-α are associated with early graft regeneration after living donor liver transplantation
Source: PLoS One. 2018 Apr 12;13(4):e0195262. doi: 10.1371/journal.pone.0195262 (PMC5896938; doi:10.1371/journal.pone.0195262)
Supplement: S1 Table — (DOCX) [file pone.0195262.s001.docx]

| **S1 Table. Correlations of preoperative serum cytokine profiles with preoperative clinical recipient findings in living donor liver transplantation**. | | | | | | | | | | | | | | | |  |
| --- | --- | --- | --- | --- | --- | --- | --- | --- | --- | --- | --- | --- | --- | --- | --- | --- |
|  | **Age** | **Gender** | **BMI** | **MELD** | **Etiology** | **Hct** | **Sodium** | **WBC** | **CRP** | **Platelet** | **T. Bil** | **INR** | **AST** | **ALT** | **Alb** | |
| **IL-2** | 0.02 | -0.10 | -0.02 | 0.07 | -0.08 | 0.02 | -0.17* | 0.01 | -0.00 | -0.04 | 0.06 | 0.02 | 0.07 | 0.02 | 0.07 | |
| **IL-6** | -0.01 | -0.00 | 0.01 | 0.27** | 0.01 | -0.15* | -0.29** | 0.26** | 0.50** | -0.02 | 0.26** | 0.22** | 0.23** | 0.21** | -0.15* | |
| **IL-10** | -0.03 | 0.02 | -0.04 | 0.19** | 0.06 | -0.17* | -0.05 | 0.22** | 0.35** | -0.07 | 0.22** | 0.12 | 0.23** | 0.20** | -0.00 | |
| **IL-12** | 0.11 | -0.12 | -0.03 | 0.08 | -0.08 | -0.11 | -0.10 | -0.03 | -0.09 | -0.02 | 0.07 | 0.08 | 0.12 | 0.07 | -0.02 | |
| **IL-17** | 0.06 | -0.10 | -0.05 | 0.01 | -0.05 | 0.00 | -0.10 | -0.01 | -0.08 | -0.02 | 0.05 | 0.05 | 0.04 | 0.06 | -0.01 | |
| **IFN-γ** | 0.05 | -0.03 | -0.02 | 0.02 | -0.03 | -0.01 | -0.11 | 0.03 | -0.03 | -0.06 | 0.05 | 0.03 | 0.00 | -0.03 | -0.11 | |
| **TNF-α** | -0.08 | 0.10 | -0.07 | 0.19** | 0.05 | -0.19** | -0.14* | 0.16* | 0.23** | 0.01 | 0.13 | 0.09 | 0.12 | 0.05 | -0.18** | |
| **Abbreviations:** IL, interleukin; IFN, interferon; TNF, tumor necrosis factor; BMI, body mass index; MELD, model for end stage liver disease; Hct, hematocrit; WBC, white blood cell count; CRP, C-reactive protein; T. Bil, total bilirubin; INR, international normalized ratio; AST, aspartate aminotransferase; ALT, alanine aminotransferase; Alb, albumin  **p* <0.05, ***p*<0.01 | | | | | | | | | | | | | | | |  |
