# Supplementary material for: Serum interleukin-6 and tumor necrosis factor-α are associated with early graft regeneration after living donor liver transplantation
Source: PLoS One. 2018 Apr 12;13(4):e0195262. doi: 10.1371/journal.pone.0195262 (PMC5896938; doi:10.1371/journal.pone.0195262)
Supplement: S2 Table — (DOCX) [file pone.0195262.s002.docx]

| **S2 Table. Comparisons of preoperative serum cytokine levels according to gender in patients who underwent living donor liver transplantation.** | | | |
| --- | --- | --- | --- |
|  | **Male** | **Female** |  |
| **Serum cytokine level (pg/mL)** | **n = 159** | **n = 67** | ***p*** |
| **Interleukin-2** | 0.1 (0.1 - 1.8) | 0.1 (0.1 - 1.0) | 0.144 |
| **Interleukin-6** | 6.7 (0.1 - 29.8) | 7.6 (0.3 - 32.4) | 0.951 |
| **Interleukin-10** | 0.5 (0.1 - 11.1) | 0.7 (0.1 - 10.1) | 0.819 |
| **Interleukin-12** | 0.1 (0.1 - 0.1) | 0.1 (0.1 - 0.1) | 0.062 |
| **Interleukin-17** | 2.3 (0.1 - 20.2) | 1.5 (0.1 - 8.6) | 0.127 |
| **Interferon-γ** | 3.2 (0.1 - 13.3) | 2.9 (0.1 - 17.9) | 0.985 |
| **Tumor necrosis factor-α** | 9.3 (5.1 - 16.7) | 12.9 (6.3 -19.6) | 0.141 |
| **NOTE:** Values are expressed as median and interquartile range. | | | |
