# Supplementary material for: Serum interleukin-6 and tumor necrosis factor-α are associated with early graft regeneration after living donor liver transplantation
Source: PLoS One. 2018 Apr 12;13(4):e0195262. doi: 10.1371/journal.pone.0195262 (PMC5896938; doi:10.1371/journal.pone.0195262)
Supplement: S3 Table — (DOCX) [file pone.0195262.s003.docx]

| **S3 Table. Comparisons of preoperative serum cytokine levels according to etiology in patients who underwent living donor liver transplantation.** | | | | | | | |
| --- | --- | --- | --- | --- | --- | --- | --- |
|  | **Etiology** | | | | | |  |
|  | **Alcohol** | **Hepatitis A** | **Hepatitis B** | **Hepatitis C** | **Drug & toxin** | **Cryptogenic** |  |
| **Serum cytokine level (pg/mL)** | **n = 44** | **n = 5** | **n = 138** | **n = 12** | **n = 15** | **n = 12** | ***p*** |
| **IL-2** | 0.1 (0.1 - 1.8) | 0.9 (0.1 - 8.7) | 0.1 (0.1 - 1.7) | 0.1 (0.1 - 0.1) | 0.1 (0.1 - 2.5) | 0.1 (0.1 - 0.1) | 0.139 |
| **IL-6** | 6.6 (3.4 - 36.7) | 9.4 (4.3 - 33.6) | 6.5 (0.1 - 28.5) | 5.9 (0.1 - 13.3) | 20.7 (3.0 - 44.3) | 8.3 (1.6 - 31.5) | 0.267 |
| **IL-10** | 1.1 (0.1 - 6.3) | 11.1 (0.1 - 35.4) | 0.3 (0.1 - 10.1) | 0.5 (0.1 - 14.0) | 11.0 (0.1 - 15.7) | 1.7 (0.1 - 6.5) | 0.504 |
| **IL-12** | 0.1 (0.1 - 0.1) | 0.1 (0.1 - 58.1) | 0.1 (0.1 - 0.1) | 0.1 (0.1 - 0.1) | 0.1 (0.1 - 0.1) | 0.1 (0.1 - 0.1) | 0.704 |
| **IL-17** | 1.4 (0.1 - 14.9) | 0.1 (0.1 - 8.4) | 3.9 (0.1 - 24.2) | 0.1 (0.1 - 3.2) | 0.1 (0.1 - 8.6) | 3.6 (0.1 - 26.1) | 0.067 |
| **IFN-γ** | 3.4 (0.1 - 16.9) | 5.6 (2.1 - 36.0) | 4.1 (0.1 - 21.2) | 0.1 (0.1 - 2.7) | 3.2 (0.1 - 9.6) | 3.7 (0.4 - 18.0) | 0.094 |
| **TNF-α** | 10.8 (5.2 - 21.8) | 16.1 (8.1 - 25.4) | 8.9 (5.0 - 15.9) | 12.5 (6.1 - 27.3) | 18.4 (5.5 - 64.9) | 13.5 (7.2 - 16.3) | 0.285 |
| **Abbreviations:** IL, interleukin; IFN, interferon; TNF, tumor necrosis factor  **NOTE:** Values are expressed as median and interquartile range. | | | | | | | |
