# Supplementary material for: Serum interleukin-6 and tumor necrosis factor-α are associated with early graft regeneration after living donor liver transplantation
Source: PLoS One. 2018 Apr 12;13(4):e0195262. doi: 10.1371/journal.pone.0195262 (PMC5896938; doi:10.1371/journal.pone.0195262)
Supplement: S4 Table — (DOCX) [file pone.0195262.s004.docx]

| **S4 Table. Comparisons of preoperative serum cytokine levels according to model for end-stage liver disease (MELD) score in patients who underwent living donor liver transplantation.** | | | | | |
| --- | --- | --- | --- | --- | --- |
|  | **MELD score (point)** | | | |  |
|  | **1-10** | **11-20** | **21-30** | **31-40** |  |
| **Serum cytokine level (pg/mL)** | **n = 74** | **n = 86** | **n = 41** | **n = 25** | ***p*** |
| **Interleukin-2** | 0.1 (0.1 - 1.6) | 0.1 (0.1 - 1.5) | 0.1 (0.1 - 1.2) | 0.1 (0.1 - 3.8) | 0.432 |
| **Interleukin-6** | 3.9 (0.1 - 11.0) | 6.6 (0.1 - 30.9) | 17.2 (5.0 - 37.0) | 19.0 (6.2 - 56.2) | 0.000 |
| **Interleukin-10** | 0.1 (0.1 - 4.7) | 0.7 (0.1 - 12.3) | 3.2 (0.1 - 18.3) | 5.8 (0.1 - 22.0) | 0.008 |
| **Interleukin-12** | 0.1 (0.1 - 0.1) | 0.1 (0.1 - 0.1) | 0.1 (0.1 - 0.1) | 0.1 (0.1 - 0.1) | 0.514 |
| **Interleukin-17** | 1.6 (0.1 - 19.0) | 1.8 (0.1 - 13.7) | 2.4 (0.1 - 13.3) | 1.5 (0.1 - 38.1) | 0.966 |
| **Interferon-γ** | 2.2 (0.1 - 15.4) | 3.2 (0.1 - 19.5) | 2.9 (0.1 - 11.0) | 6.2 (0.2 - 23.3) | 0.524 |
| **Tumor necrosis factor-α** | 8.3 (4.0 - 14.5) | 10.5 (5.6 - 18.7) | 10.8 (5.0 - 19.6) | 15.9 (8.7 - 42.2) | 0.011 |
| **NOTE:** Values are expressed as median and interquartile range. | | | | | |
