# Supplementary material for: Serum interleukin-6 and tumor necrosis factor-α are associated with early graft regeneration after living donor liver transplantation
Source: PLoS One. 2018 Apr 12;13(4):e0195262. doi: 10.1371/journal.pone.0195262 (PMC5896938; doi:10.1371/journal.pone.0195262)
Supplement: S5 Table — (DOCX) [file pone.0195262.s005.docx]

| **S5 Table. Comparisons of preoperative serum cytokine levels according to acute cellular rejection development after living donor liver transplantation.** | | | |
| --- | --- | --- | --- |
|  | **Acute cellular rejection** | |  |
|  | **No** | **Yes** |  |
| **Serum cytokine level (pg/mL)** | **n=167** | **n=59** | ***p*** |
| **Interleukin-2** | 0.1 (0.1 - 1.4) | 0.1 (0.1 - 3.2) | 0.279 |
| **Interleukin-6** | 7.6 (0.1 - 28.1) | 6.4 (1.2 - 35.5) | 0.957 |
| **Interleukin-10** | 1.0 (0.1 - 12.2) | 0.3 (0.1 - 7.1) | 0.637 |
| **Interleukin-12** | 0.1 (0.1 - 0.1) | 0.1 (0.1 - 0.1) | 0.213 |
| **Interleukin-17** | 1.7 (0.1 - 14.8) | 2.3 (0.1 - 28.6) | 0.305 |
| **Interferon-γ** | 2.9 (0.1 - 12.6) | 5.3 (0.1 - 28.7) | 0.202 |
| **Tumor necrosis factor-α** | 9.9 (5.0 - 18.4) | 9.0 (5.6 - 16.3) | 0.789 |
| **NOTE:** Values are expressed as median and interquartile range. | | | |
